# A post-discharge pharmacist clinic to reduce hospital readmissions: a retrospective cohort study

**DOI:** 10.1007/s11096-025-01923-1

**Published:** 2025-04-26

**Authors:** Jaclyn Costello, Michael Barras, Centaine L. Snoswell, Holly Foot

**Affiliations:** 1 The School of Pharmacy, The University of Queensland, 20 Cornwall Street, Woolloongabba, Brisbane, QLD 4102 Australia; 2 Pharmacy Department, Redcliffe Hospital, Brisbane, QLD Australia; 3 Pharmacy Department, Princess Alexandra Hospital, Brisbane, QLD Australia; 4 Centre for Online Health, Centre for Health Services Research, The University of Queensland, Brisbane, QLD Australia

**Keywords:** Hospital readmissions, Patient clinical outcomes, Pharmaceutical care, Pharmacist, Post-discharge medication review

## Abstract

**Background:**

Patients transitioning from secondary to primary healthcare are at increased risk of medication errors, adverse drug events and readmission to hospital. Incorporating a post-discharge follow-up by a hospital pharmacist has been proposed as a potential strategy to reduce readmissions.

**Aim:**

To determine the impact of a hospital-based pharmacist-led post-discharge medication review clinic on 30-day hospital readmissions in adult patients.

**Method:**

A single-site, retrospective cohort study compared the medical records of patients who attended the Pharmacist Review and EValuation of Existing and New Therapies (PREVENT) clinic between 1 January 2018 and 31 December 2019 to a group of case-matched control patients who did not attend the clinic. Patient inclusion criteria comprised those 18 years and older and attended the PREVENT clinic within 30 days of discharge. The matched group was based on gender, age and hospital metrics. The primary outcome measure is unplanned, all-cause 30-day hospital readmission.

**Results:**

There were 170 patients per group, with similar baseline characteristics. There were significantly less unplanned all-cause 30-day hospital readmissions in the PREVENT clinic group (n = 12 (7.1%)) compared to the control group (n = 40 (23.5%), χ^2^ = 17.799, *p* < 0.001).

**Conclusion:**

This study demonstrates that a hospital-based pharmacist-led post-discharge medication review clinic reduced 30-day hospital readmissions in adult patients compared to a group of case-matched controls. This study provides evidence to support extending pharmaceutical care beyond the inpatient hospital setting into the early post-discharge period, particularly in hospitals providing comprehensive clinical pharmacy services.

**Supplementary Information:**

The online version contains supplementary material available at 10.1007/s11096-025-01923-1.

## Impact statements

- Improving medication management, medication errors and adverse drug events at transitions of care from hospital to primary healthcare with pharmacist-led post-discharge medication review is likely to improve patient outcomes and health service metrics, particularly for patients with chronic disease and those at high risk of medication misadventure.
- Providing post-discharge medication review in a hospital-based clinic in an Australian hospital setting significantly reduced 30-day hospital readmissions compared to comprehensive inpatient pharmaceutical care alone. This service model allows patients to access timely post-discharge medication review, improves communication between hospital and primary care as well as improves patient outcomes.
- Health services and hospitals internationally that provide comprehensive inpatient clinical pharmacy services may improve patient outcomes, such as 30-day hospital readmissions, by adding follow-up in a pharmacist-led post-discharge medication review clinic to their standard of care.

## Introduction

Patients transitioning between secondary and primary health care are at increased risk of medication errors, adverse drug events (ADEs), medication-related problems (MRPs) and readmission to hospital [1–4]. Rates of readmission due to medications and ADEs has been reported to be between 3 and 64% (median 21%), with between 5 and 87% (median 69%) of these potentially being preventable [5]. Inpatient pharmaceutical care provided by a pharmacist has been shown to reduce medication errors, ADEs, length of stay (LOS) and post-hospital healthcare utilisation [6–10]. Incorporating post-discharge follow-up by a pharmacist into the transitions of care model has been proposed as a potential way to further reduce readmission to hospital [11–13].

A systematic review of the impact of a hospital-based pharmacist-led post-discharge medication review on patient clinical outcomes revealed that there were three main outcome metrics commonly reported. These were hospital readmission and/or representation (n = 45), adverse events (n = 12) and improved disease state metrics (n = 2) [14]. The review revealed that overall a post-discharge clinic pharmacist improves patient clinical outcomes, most commonly 30-day hospital readmissions [14]. The impact of post-discharge pharmacist review on 30-day readmissions has also been explored and reported to be effective for improving this outcome in multi-study analyses by Rodrigues et al. and Weber et al. [15, 16]

Hospital clinical services are expanding rapidly as evidence for pharmacist-led pharmaceutical care throughout transitions of care and after hospital discharge continues to build [8, 9, 14–19]. Despite research supporting pharmacist-led post-discharge care [8, 9, 14–16, 18, 20], there is little evidence in an Australian healthcare setting assessing the impact of hospital-based clinical pharmacy services for patients in the early post-discharge period. The systematic review identified 57 studies, none of which were Australian [14], and it was difficult to determine whether the studies identified provided an inpatient model of care similar to gold standard or comprehensive clinical pharmacy services (usual care) that is expected to be provided in hospital in Australia [1, 14, 21, 22].

At a metropolitan hospital, the Pharmacist Review and EValuation of New and Existing Therapies (PREVENT) clinic, was implemented with an aim to improve medicine management after discharge from hospital.

The purpose of this study was to evaluate the impact of a hospital-based pharmacist-led post-discharge medication review clinic, the PREVENT clinic, on patient clinical outcomes including 30-day hospital readmissions.

### Aim

To determine the impact of a hospital-based, pharmacist-led post-discharge medication review clinic on 30-day hospital readmissions in adult patients.

### Ethics approval

Ethics approval was received from the Metro North Health Human Research Ethics Committee (Approval: HREC/2020/QRBW/65110).

## Method

This study was a single-site, retrospective cohort study. A retrospective review of medical records was conducted for all patients who attended the PREVENT clinic between 1 January 2018 and 31 December 2019 at an Australian metropolitan hospital. The group of case-matched control patients did not attend the clinic and received usual care at the same hospital.

### The prevent clinic service (Intervention group)

The PREVENT clinic commenced in September 2016 to facilitate timely post-discharge follow-up by a pharmacist targeting patients at risk of medication misadventure, [23, 24] meaning those at risk of medication misuse or medication harm and early readmission to hospital. The PREVENT clinic service was modelled on the Home Medicines Review (HMR) service in Australia, which aims to support the quality use of medicines, reduce MRPs or healthcare use and help patients better understand their medicines [25–27].

Patients were referred to the PREVENT clinic by a member of the inpatient treating team who were identified to be at risk of medication misadventure. A member of the treating team included a doctor, pharmacist, nurse, or other health professional. The key patient criteria for referral to the PREVENT clinic included taking five or more regular medications, taking a high-risk medication, one or more medication changes during admission, identified to be non-adherent with medication therapy or having difficulty managing their medicines, and/or requiring further education and follow-up. High risk medications were defined as medicines that have an ‘increased risk of causing significant patient harm if they are misused or used in error’ as defined by the Australian Commission on Safety and Quality in Health Care [28].

At the clinic appointment, a senior hospital pharmacist undertook a comprehensive medication review including, confirmation of medication history and medication understanding, reconciliation of current medicines with the recent hospital discharge medication record (DMR), review of each medication for safety and effectiveness, as well as identified and rectified MRPs. These MRPs were resolved directly with the patient through education or negotiation, implementation of dose administration aids if indicated, referral to another health practitioner (e.g. diabetes educator) or sent to the patient’s general practitioner (GP) via facsimile letter to optimise their medicines or disease state management. These processes are based on professional practice standards [22, 29]. Each interaction with the patient was individualised based on their needs.

The clinic appointment occurred in the hospital outpatient department either face to face, by phone or telehealth as close to the time of discharge as possible, ideally within 14 days. Each initial appointment consisted of a 45 to 60 min consultation with the patient, with documentation and GP follow-up completed after each consultation. Further follow-up with individual patients could be arranged as needed. For the purposes of this study, only the initial post-discharge review consultation has been analysed.

### Usual care (Control group)

No post-discharge care was provided by the clinic pharmacist at the study hospital. All patients received standard inpatient pharmaceutical care services as outlined in professional practice standards [22, 29]. This included undertaking a best possible medication history, medication reconciliation between the medicines taken at home and current inpatient medication chart and treatment plan, medication review of all active medical problems and treatments, identification and resolution of MRPs, discharge medication reconciliation, provision of a DMR and patient education [22, 29].

### Outcome measures

The primary outcome measured was unplanned, all-cause 30-day hospital readmission, which is internationally recognised as a key outcome of interest [15, 16, 30, 31]. Secondary outcomes included unplanned all-cause 30-day Emergency Department (ED) representation, a composite of all-cause 30-day hospital readmission and/or ED representation and total hospital readmissions and/or ED presentations 12 months after the index admission. The term representation is used to describe a non-admitted, unplanned hospital presentation (e.g. ED representation without admission). Day zero was measured as the date of discharge of the index admission and readmission and/or representation dates were collected from The Viewer, a statewide database of public hospital admissions and ED presentations available to hospital clinicians and GPs in Queensland. The dates taken from The Viewer accounted for an entire episode of care where patients may have transferred to or from another facility for specialist care not available at the facility of patient presentation.

### Patient inclusion

The intervention group included admitted patients who were 18 years or older, lived independently and were seen in the PREVENT clinic within 30-days of discharge from hospital. Case-matched control patients were selected on a 1-to-1 basis with each patient in the intervention group based on gender, age (± 2–5 years), unit of admission, index admission discharge date (± 30–60 days), and LOS or discharge related group (DRG). These criteria were chosen as patient demographics that can contribute to the risk of readmission and were able to be defined by the hospital administration database [12, 32, 33]. Index admission is the term used to designate the admission to hospital immediately preceding the PREVENT clinic appointment or the prior admission if evidence of referral was identified in an earlier admission in the medical record. The term DRG refers to the primary diagnosis as the cause of admission.

Patients who lived in a nursing home, had previously attended the PREVENT clinic, failed to attend the PREVENT clinic appointment or readmitted to hospital prior to their PREVENT clinic appointment (and their case matched control) have been excluded. Patients identified to be a non-PREVENT clinic review or patients seen by another outpatient pharmacist clinic from the clinical notes were also excluded.

### Sample size

A sample size calculation for the primary outcome of unplanned, all-cause 30-day hospital readmission, was undertaken based on results from a pre-study audit of preliminary data comparing PREVENT clinic review patients with a second group of patients who had been referred to the clinic but failed to attend an appointment [34]. This produced a sample size of 185 patients per group, based on a 28-day readmission rate of 21.75% in the ‘not seen’ group vs 10.75% in the ‘PREVENT clinic’ group, using a two-sided Z-test with pooled variance with alpha of 5% and power of 80%. The difference in readmission rate observed in this audit data was similar to the 30-day readmission reduction reported in a study by Budlong et al. for their elevated, and higher risk groups [30].

### Data collection

Data were collected by the principal investigator (JC) between 1st July 2022 and 30th June 2023, who reviewed hospital databases and individual patient medical records. Data was entered into REDCap [35, 36], an online database system stored on the hospitals secure server. The full list of data collected and their corresponding sources is outlined in Appendix A.

### Data analysis

Demographic data were summarised using descriptive statistics, including frequencies and percentages for categorical variables. Continuous variables have been summarised as mean and standard deviation or median and interquartile range. Independent t-test or Mann Whitney U test (if data was not normally distributed) has been used to compare means/medians between groups, respectively. Pearson Chi-squared with Fisher’s exact test has been used to compare categorical outcome measures between the two groups. P values less than 0.05 are considered significant. Missing data or missing values were excluded from the analysis.

The disease states presented are commonly compared in the literature or represent the chronic diseases targeted for referral to the PREVENT clinic in the concept brief. The pharmaceutical care received by all patients was collected from the medical record and compared to test homogeneity of usual care received. All data analysis was conducted using SPSS Statistics® software version 29.0.1.0.

## Results

**Fig. 1:**
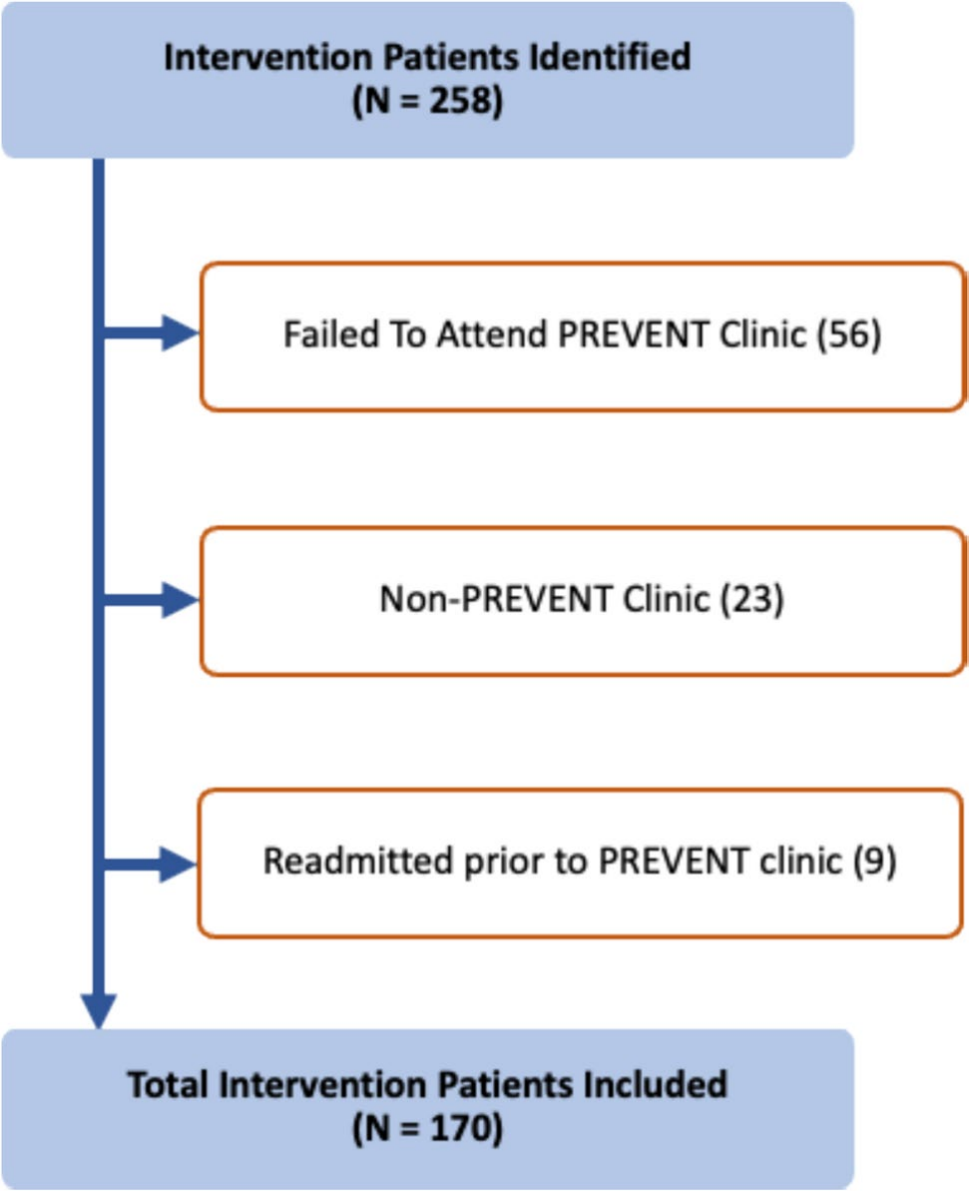
Flow diagram of patient selection for the intervention group

A total of 258 patients attended the PREVENT clinic for a post-discharge medication review, and 170 patients were suitable for the study based on inclusion and exclusion criteria (Fig. 1). These patients were case-matched to 170 controls.

### Baseline characteristics

Overall, there was no statistically significant difference in baseline characteristics between the intervention and control groups with the exception of a history of ischaemic heart disease (IHD) and uncomplicated diabetes (Table 1). There were more patients with IHD in the control group (*p* = 0.017), and more patients with uncomplicated diabetes in the intervention group (*p* < 0.001). The number of previous hospitalisations in the 12 months prior to the index admission and ADRs recorded at admission were not significantly different between the two groups.

**Table 1:** Baseline characteristics of patient population

Characteristic		Intervention N = 170	Usual Care (Control) N = 170	*p*-value^a^
Age (Mean, SD)		69.8 (14.2)	69.7 (14.2)	0.954
Gender (Female) N (%)		75 (44.1%)	75 (44.1%)	1.000
Indigenous (Aboriginal and Torres Strait Islander) N (%)		5 (2.9%)	4 (2.4%)	0.735^b^
Unit at Index Admission N (%)	Medical	124 (72.9%)	125 (73.5%)	0.841
	Surgical	28 (16.5%)	30 (17.6%)	
	Rehabilitation	18 (10.6%)	15 (8.8%)	
LOS index admission (days) (Mean, SD)		7.7 (14.9)	6.4 (13.7)	0.424
Body Mass Index (BMI) (Mean, SD)^c^		28.9 (7.4)	30.0 (8.9)	0.332
Charlson Comorbidity Index (CCI) (Mean, SD)		6.0 (2.6)	6.5 (3.1)	0.132^d^
Prior hospitalisations (12 months) (Mean, SD)		1.3 (1.7)	1.8 (3.2)	0.068^d^
Prior ED presentations (12 months) (Mean, SD)		1.8 (2.7)	2.4 (4.5)	0.149^d^
Number of ADRs (medications) at admission (Mean, SD)^e^		1.1 (1.9)	1.2 (1.8)	0.677
Number of prescription medications at discharge (Mean, SD)^f^		10.8 (4.7)	8.8 (5.0)	0.353
Chronic Conditions N (%)	Hypertension	109 (64.1%)	92 (54.1%)	0.061
	IHD	39 (22.9%)	59 (34.7%)	0.017
	CCF	36 (21.2%)	29 (17.1%)	0.334
	AF	48 (28.2%)	44 (25.9%)	0.625
	COPD	33 (19.4%)	43 (25.3%)	0.193
	Asthma	23 (13.5%)	32 (18.8%)	0.185
	Diabetes (uncomplicated)	56 (32.9%)	25 (14.7%)	< 0.001
	Diabetes (end-organ complications)	15 (8.8%)	17 (10.0%)	0.710
	Mod-severe CKD	19 (11.2%)	24 (14.1%)	0.415
	CVA/TIA	29 (17.1%)	24 (14.1%)	0.455
	Chronic pain	22 (12.9%)	23 (13.5%)	0.600^b^
	Dementia	4 (2.4%)	9 (5.3%)	0.157

^a^Unless otherwise noted, Student’s t-test was used for continuous variables (mean, SD) and the Pearson χ^2^ test was used for categorical variables (N, %)

^b^2 cells have expected count less than 5

^c^N = 112 (intervention), N = 115 (control)

^d^Welch’s t-test was used for these variables

^e^N = 169 (intervention), N = 170 (control)

^f^N = 155 (intervention), N = 162 (control)

*ADRs* Adverse Drug Reactions, *AF* Atrial Fibrillation, *BMI* Body Mass Index, *C* Control, *CCF* Congestive Cardiac Failure, *CCI* Charlson Comorbidity Index, *CKD* Chronic Kidney Disease, *COPD* Chronic Obstructive Pulmonary Disease, *CVA* Cerebrovascular Accident, *ED* Emergency Department, *I*  Intervention, *IHD* Ischaemic Heart Disease, *LOS* Length of Stay, *MST* Malnutrition Screening Tool, *SD* Standard deviation, *TIA* Transient Ischaemic Attack

### Baseline pharmaceutical care received during index admission

There was no significant difference in the number of patients who had a best possible medication history taken and the number of discharge summaries completed between the two groups (Table 2). There were signficantly more patients in the intervention group who received medication reconciliation, a DMR, discharge medications included in the discharge summary and a complete Pharmaceutical Care Bundle (PCB) [6, 37], compared to the control group. All patients had a discharge summary completed.

**Table 2:** Baseline pharmaceutical care received by patients during index admission

Inpatient clinical pharmacist activities	Intervention N = 170 (%)	Control N = 170 (%)	*p*-value^a^
Best possible medication history	164 (96.5%)	158 (92.9%)	0.146
Medication reconciliation (admission)	152 (89.4%)	139 (81.8%)	0.045
Discharge Medication Record (DMR) provided	156 (91.8%)	135 (79.4%)	0.001
Discharge medications included in discharge summary	157 (92.4%)	143 (84.1%)	0.022
Pharmaceutical Care Bundle (received all four activities)	138 (81.2%)	113 (66.5%)	0.002
Discharge summary completed	164 (96.5%)	164 (96.5%)	1.000

^a^Pearson χ^2^ test

### The PREVENT clinic intervention results

The mean (± SD) number of days from discharge to PREVENT clinic appointment was 14.9 (± 7.0), ranging from 2 to 29 days. Of the 170 patients seen in the PREVENT clinic, the progress notes from the PREVENT clinic review could not be located for seven patients. For the remaining 163 patients’ clinic progress notes analysed, there was a mean of 4.6 (± 1.7) pharmacist interventions documented per patient, ranging from one to nine interventions in total. A pharmacist intervention could have included patient education, referral to another health practitioner or recommendation regarding optimisation of medication or treatment for the prescriber to review.

### Outcomes

#### Primary outcome

There were 12 (7.1%) patients readmitted within 30-days from index discharge in the intervention group and 40 (23.5%) in the control group (Table 3). The primary outcome, unplanned all-cause 30-day hospital readmission was significantly lower in the intervention group compared to the control group (χ^2^ = 17.799, *p* < 0.001).

**Table 3:** All-cause 30-day hospital readmissions, 30-day ED representations and composite 30-day readmission and/or ED representation (n = 170)

	Intervention (PREVENT clinic)	Control	*p*-value^a^
30-day readmission	12 (7.1%)	40 (23.5%)	< 0.001
30-day ED representation (non-admitted)	7 (4.1%)	10 (5.9%)	0.455
Composite 30-day readmission and/or ED representation	19 (11.2%)	50 (29.4%)	< 0.001

^a^Pearson χ^2^ statistic

#### Secondary outcomes

A total of 7 (4.1%) patients represented to ED within 30-days from index discharge in the intervention group and 10 (5.9%) in the control group, which was not significant (Table 3).

A total of 19 (11.2%) patients were readmitted and/or represented to ED within 30-days from index discharge (composite 30-day readmission and/or ED representation) in the intervention group and 50 (29.4%) in the control group (χ^2^ = 17.474, *p* < 0.001, Table 3). The total number of readmissions and ED representations per patient in the 12 months after discharge from the index admission is summarised in Table 4. There was no difference between the two groups.

**Table 4:** Total readmissions and/or ED representations after index admission

Measure	Intervention (N = 170)			Control (N = 170)			*p*-value^a^
	Range	Mean	Standard deviation	Range	Mean	Standard deviation	
Readmissions–12 months	0–17	1.56	2.430	0–18	1.69	2.479	0.321
ED representations–12 months	0–21	1.82	3.041	0–19	1.97	3.080	0.323

^a^Student’s t-test (one-sided)

## Discussion

This study demonstrated that the addition of post-discharge follow-up by a hospital-based clinic pharmacist providing comprehensive medication review in addition to comprehensive inpatient pharmaceutical care significantly reduced unplanned, all-cause 30-day hospital readmission, when compared to case matched controls. The intervention arm consisted of patients who received a comprehensive medication review in the PREVENT clinic by a pharmacist who identified and resolved MRPs by liaising with patients, their GP, the hospital treating team or referral to other healthcare professionals in addition to usual inpatient pharmaceutical care. These results support other studies that demonstrate the importance of comprehensive medication review in the post-discharge period that includes direct communication with the primary care provider as a key feature of post-discharge pharmaceutical care likely to reduce hospital readmission, especially at 30-days [10, 15, 16, 20, 30, 31, 38, 39].

In the United States (US) similar post-discharge pharmacist services that include comprehensive mediation reviews, physician collaboration, and shared health records reduced readmissions by 8–32% [30, 39, 40]. Medication therapy management (MTM) or comprehensive medication management (CMM) services in the US are similar to the medication management review program for the undertaking of HMRs here in Australia [18, 25], which the PREVENT clinic medication review process is based. The key difference between CMM and a HMR or the PREVENT clinic comprehensive medication review, is that a CMM review is undertaken in collaboration with one or more physicians in accordance with defined protocols utilising a shared medical record [41]. Additionally, the pharmacist is able to order laboratory tests, assess medication effectiveness and adjust medications in accordance with those protocols [41]. This direct intervention and resolution approach is likely to be more efficient for resolving MRPs than the HMR or PREVENT clinic process in Australia.

Post-discharge medication review has always been a priority referral criteria for a HMR in Australia [25]. However, various barriers such as timely access to a HMR and time pressures on hospital pharmacists have been identified as key reasons a post-discharge HMR has not yet become common practice [42]. In Australia there is limited evidence exploring post-discharge medication review on patient outcomes in the literature [38, 43, 44]. Our clinic is able to provide a similar service to a HMR or GP practice pharmacist with the additional benefit of being able to access inpatient records and test results that may not be detailed in the discharge summary, with improved efficiency by eliminating travel time to patients homes. This study provides further evidence to support a comprehensive medication review service by a pharmacist in the post-discharge period, particularly for patients at high risk of experiencing MRPs or ADEs.

There have been systematic reviews published in which the study authors have proposed that several components of pharmaceutical care from a pharmacist may best influence patient outcomes such as hospital readmission [9, 13, 14, 45, 46]. The components likely to be responsible for these effects include medication reconciliation, medication review and patient education, that is incorporated into inpatient pharmaceutical care by a pharmacist in hospital, as well as additional post-discharge comprehensive medication review [9, 13, 14, 45, 46].

The results of our study comparing a complete PCB by a pharmacist during the index admission was higher for patients in the intervention group, which may have contributed to the observed reduction in 30-day hospital readmission [6]. However, it is to be expected that intervention patients received this enhanced pharmaceutical care or a complete PCB by the pharmacist during their admission as they were identified to be at high risk of medication misadventure and referred to the PREVENT clinic for post-discharge follow-up. And despite this potential improved inpatient pharmaceutical care, there was still an average of 4.6 MRPs and/or ADEs identified per patient by the PREVENT clinic pharmacist at their post-discharge review appointment. This suggests that optimisation of the patient’s overall health was not completed during the index admission.

What remains unclear is which specific pharmaceutical care activities or at which point in the patients transition of care may best influence patient outcomes [14]. What is becoming evident is that providing patients with the ability to access post-discharge pharmaceutical care in their home, primary care or a hospital-based setting with direct communication with their GP seems to be important for improving patient outcomes.

Future research to examine the long-term effects, cost-effectiveness of the service and identification of patient risk factors for readmission to target services to those most likely to benefit from a post-discharge pharmacy clinic is needed to help prioritise healthcare services and healthcare spending to best influence patient outcomes.

### Limitations and control of bias or confounding

Bias and confounding were reduced through close matching of control group patients with the intervention group by gender, age, unit of admission, date of initial hospitalisation, LOS and/or DRG. Key risk factors for hospital admission and readmission such as age, LOS and DRG were chosen with the aim of ensuring each group had the same chance of hospital readmission. At the time of writing the study protocol it was not known if it was possible to match the control patients based on number of previous hospital admissions, hence this criteria was not used for generating the control group. Outcome variables may have been affected if a patient was not readmitted to a Queensland public hospital (e.g. a private hospital or interstate) or died, due to the information systems utilised for data collection.

Patients were not matched based on inpatient pharmaceutical care received during their index admission. At the time of designing this study it was not thought that this data would be readily available from hospital databases to enable this closer matching of usual care services received but may have strengthened the results observed. Since designing this study the paper by Canning et al. has been published which shows that a complete PCB received by patients during their inpatient stay by a hospital pharmacist reduces their risk of readmission [6]. However, we do not think that the results observed with regard to the inpatient pharmaceutical care received in the two groups is solely responsible for the difference in 30-day readmission observed in this study, and that post-discharge pharmaceutical care in addition to comprehensive inpatient pharmaceutical care is needed to positively affect 30-day hospital readmissions.

Limitations or bias which may have occurred from excluding patients who readmitted to hospital prior to their PREVENT clinic appointment and their case-matched control was thoroughly debated by the research team. However, this was not evident when an evaluation of the outcomes that included these additional nine patients was performed, which also produced a statistically significant difference in 30-day readmission (Appendix B).

## Conclusion

Our study shows post-discharge medication review that addresses MRPs with the patient’s care team reduced 30-day hospital readmission compared to usual care. This collaborative model of care is likely to benefit patients with chronic disease and/or multiple chronic conditions by utilising the skills of a pharmacist to aid in the optimising of their medication management, identification of MRPs and ADEs and improve monitoring of medication effectiveness or potential harm. This study provides evidence to support extending pharmaceutical care beyond the inpatient setting into the early post-discharge period, particularly where comprehensive clinical pharmacy services are provided in hospital.

## Supplementary Information

Below is the link to the electronic supplementary material.

Supplementary file1 (DOCX 34 kb)
