# Supplementary material for: A post-discharge pharmacist clinic to reduce hospital readmissions: a retrospective cohort study
Source: Int J Clin Pharm. 2025 Apr 26;47(5):1315–23. doi: 10.1007/s11096-025-01923-1 (PMC12431875; doi:10.1007/s11096-025-01923-1)
Supplement: Supplementary file 1 — Supplementary file1 (DOCX 34 kb) [file 11096_2025_1923_MOESM1_ESM.docx]

# Supplementary material

## Appendix A: Full list of data collection sources

| **Data collected** | **Data Source** | **Group 1** | **Group 2** |
| --- | --- | --- | --- |
| Unique Record Number (URN) (see Data Management) | HIU | ✔ | ✔ |
| Date of Birth / Age (years) at index admission | HIU | ✔ | ✔ |
| Sex | HIU | ✔ | ✔ |
| Date of index admission | HIU | ✔ | ✔ |
| Date of index discharge | HIU | ✔ | ✔ |
| Length of stay during index admission (days) | HIU | ✔ | ✔ |
| Presenting Complaint/Provisional Diagnosis at index admission | PR/ICD-10 | ✔ | ✔ |
| Co-morbidities (Medical Conditions) at discharge of index admission | PR/ICD-10 | ✔ | ✔ |
| New diagnoses at index admission | PR/ICD-10 | ✔ | ✔ |
| Concomitant medications at admission (index admission) | PR/eLMS | ✔ | ✔ |
| Concomitant medications at discharge (index admission) | PR/eLMS | ✔ | ✔ |
| Concomitant medications at PREVENT Clinic review | PR/eLMS | ✔ |  |
| Time since discharge from index admission (days) to PREVENT clinic appointment | HIU | ✔ |  |
| Hospital readmission within 30-days of discharge (yes/no) | HIU/Viewer | ✔ | ✔ |
| ED presentation within 30-days of discharge (yes/no) | HIU/Viewer | ✔ | ✔ |
| Date/number of any subsequent statewide public hospital admission(s) (within 12 months of discharge from index admission)^a^ | HIU/Viewer | ✔ | ✔ |
| Time since discharge from index admission (days) and any (state-wide public hospital) subsequent admission or ED presentation (up to 12 months following index admission)^a^ | Calculated | ✔ | ✔ |
| Concomitant medications at admission (subsequent readmission only if within 30 days of discharge of index admission) | PR/eLMS | ✔ | ✔ |
| Concomitant medications at discharge (subsequent readmission only if within 30 days of discharge of index admission) | PR/eLMS | ✔ | ✔ |
| Relevant biochemical parameters (sodium, potassium, magnesium, SCr, eGFR, LFTs, INR, APTT) at index admisison | Auslab | ✔ | ✔ |
| Pharmaceutical care activities provided by a pharmacist^b^: |  |  |  |
| - During their index admission | PR | ✔ | ✔ |
| - During their PREVENT clinic review | PR | ✔ |  |
| MRPs/ADEs identified: |  |  |  |
| - During their PREVENT clinic review | PR | ✔ |  |
| - During subsequent readmission (if within 30 days of index) | PR/Viewer | ✔ | ✔ |
| Data related to 30-day readmission events for patient cohorts: |  |  |  |
| - Date of admission and discharge (confirmed against The Viewer) | HIS/HIU or casemix | ✔ | ✔ |
| - New diagnoses at readmission (DRG, principle diagnosis and principle diagnosis description) | HIS/HIU or casemix | ✔ | ✔ |
| - Length of stay during readmission (days) | HIS/HIU or casemix | ✔ | ✔ |
| - ICD-10 codes, diagnosis_sequence, diagnosis, ICD long description (ICD_LONG_DESC) | HIS/HIU or casemix | ✔ | ✔ |
| - Cost of admission | HIS/HIU or casemix | ✔ | ✔ |
| - Care type or acute/non-acute designation | HIS/HIU or casemix | ✔ | ✔ |
| Bed day average cost for acute or non-acute admissions for financial years: 2017-18, 2018-19, 2019-20 | HIS/HIU, casemix, or business unit | ✔ | ✔ |
| Intervention costs (including: staff, information technology and other resources) for financial years: 2017-18, 2018-19, 2019-20 | Business Unit / Expert Opinion | ✔ | ✔ |

ADE = Adverse Drug Event; APTT = Activated Partial Thromboplastin Time; ED = Emergency Department; eGFR = Estimated Glomerular Filtration Rate; eLMS = Enterprise Liaison Medication System; HIU = Health Information Unit; HIS = Health Information Service; ICD-10 = International Classification of Diseases – Tenth Revision; INR = International Normalised Ratio; LFTs = Liver Function Tests; MRP = Medication-related Problem; PR = Patient Record; PREVENT = Pharmacist Review and Evaluation of Existing and New Therapies; SCr = Serum Creatinine.

^a^Evidence of readmission during 12-months following discharge. A 12-month period for readmission was chosen to capture long-term effects from a pharmacist intervention, as suggested by Kwan et al.[40]

^b^A pharmaceutical care activity will include:

- A best possible medication history (BPMH)
- A medication management assessment (e.g. adherence, use of dose administration aids, swallowing difficulties) if documented
- Medication reconciliation (if documented)
  - At admission
  - At discharge
- Pharmaceutical review, including:
  - Identification of MRPs or ADEs
  - Interventions made regarding MRPs or ADEs and their outcome (if documented)
  - Medication optimisation
- Provision of a Discharge Medication Record (DMR)
- Patient education provided

## Appendix B: Evaluation of 30-day outcomes with readmitted patients included (Intention to treat).

An evaluation of outcomes that included the additional 9 patients who readmitted prior to the PREVENT clinic appointment and their case-matched controls (intention to treat analysis) demonstrated similar results. The PREVENT clinic significantly reduced 30-day readmission (n = 21 (11.7%) vs 42 (23.5%), p = 0.004) and composite 30-day readmission and/or representation (n = 29 (16.2%) vs 54 (30.2%), p = 0.002). A non-significant reduction in 30-day ED representation (n = 8 4.5%) vs 12 (6.7%), p = 0.357) was observed in the intervention group compared to the control group.

### Table All-cause 30-day hospital readmissions, 30-day ED representations and composite 30-day readmission and/or ED representation (n = 179).

|  | **Intervention  (PREVENT clinic)** | **Control** | **p-value^a^** |
| --- | --- | --- | --- |
| 30-day readmission | 21 (11.7%) | 42 (23.5%) | 0.004 |
| 30-day ED representation (non-admitted) | 8 (4.5%) | 12 (6.7%) | 0.357 |
| Composite 30-day readmission and/or ED representation | 29 (16.2%) | 54 (30.2%) | 0.002 |
| ^a^Pearson χ^2^ statistic. |  |  |  |
